# TNBC vs. Non-TNBC: A Five-Year Retrospective Review of Differences in Mean Age, Family History, Smoking History and Stage at Diagnosis at an Inner City University Program

**DOI:** 10.4021/wjon738w

**Published:** 2014-01-16

**Authors:** Khurram Tariq, Fauzia Rana

**Affiliations:** a Department of Internal Medicine, College of Medicine, University of Florida, Jacksonville, FL 32209, USA; b Division of Hematology & Medical Oncology, College of Medicine, University of Florida, Jacksonville, FL 32209, USA

**Keywords:** TNBC, Non-TNBC, Breast cancer, Demographical differences

## Abstract

**Background:**

In recent years, breast cancer has been classified on the basis of estrogen or progesterone receptor (ER/PR) status and whether the human epidermal growth factor 2 receptor (HER2/neu) protein is overexpressed. Based on this system, breast cancer is broadly divided into the triple negative breast cancer (TNBC) and the non-TNBC subtypes. TNBC is a subtype of breast cancer, notable for its propensity to metastasize early and display a comparatively more aggressive course than its non-TNBC counterpart. Certain clinico-pathologic and demographic risk factors have been associated with breast cancer. In this study, we aim to compare mean age, ethnicity, family history, tobacco use and stage at presentation between TNBC and non-TNBC subtypes at our inner city university program.

**Methods:**

We reviewed data in our tumor registry between January 2000 and December 2005 with particular attention to mean age, race, family history, tobacco use and stage at presentation. We found a total of 445 patients with various subtypes of breast cancers. We included only those patients in whom the status of both ER/PR and the status of Her2/neu protein overexpression were recorded. Our strict selection criteria lead to an exclusion of about 103 patients. Out of the remaining 342 patients, 39 were TNBC and 303 were non-TNBC.

**Results:**

Mean age of onset for TNBC vs. non-TNBC patients was 59.87 ± 15.67 years vs. 60.09 ± 13.98 years respectively (P = 0.9272). In terms of ethnicity, TNBC vs. non-TNBC patients had the following racial backgrounds: black, 58.97% vs. 39.27%; white, 35.90% vs. 57.76%; Chinese, 2.56% vs. 0.99%; others, 2.57% vs. 1.98% respectively (P = 0.004, OR = 2.755). Comparisons with respect to a history of tobacco abuse for TNBC vs. non-TNBC patients revealed a positive smoking history in 20.51% vs. 27.72% whereas there was no former or current smoking history in 71.79% vs. 61.72% respectively (P = 0.4385). Comparison of family history of a breast cancer in TNBC vs. non-TNBC patients showed that positive family history of breast cancer was seen in 30.77% vs. 33.33%, no family history of cancer was seen in 51.28% vs. 51.82% and unknown 17.95% vs. 14.85% (P = 0.8384). Pathologic stage at the time of diagnosis for TNBC vs. non-TNBC patients was as follows: stage 0, 15.79% vs. 11.37% (P = 0.4332); stage 1, 34.21% vs. 30.98% (P = 0.6890); stage 2, 28.98% vs. 37.25% (P = 0.3205); stage 3, 18.42% vs. 17.25% (P = 0.0.8591); and stage 4, 3.63% vs. 3.14% (P = 0.8651). Analysis using Chi-square test revealed χ^2^ value of 0.855.

**Conclusion:**

Our results add to the growing body of evidence pertaining to the association of certain demographic and clinico-pathologic characteristics in women with breast cancer. We found that in our patient population, there is a significant ethnic predisposition for the two types of breast cancers that we studied. African Americans were more likely to have TNBC compared to the higher frequency of non-TNBC in white females. We did not find a significant difference in mean age, cigarette smoking, family history and stage at diagnosis between the TNBC and non-TNBC breast cancer patients. These findings are all consistent with the previously published research studies.

## Introduction

Breast cancer in one of the most common form of cancer among women and by some accounts it represents about a quarter of the 1.1 million newly diagnosed female malignancies each year [1, 2]. Breast cancer is also the leading cause of cancer-related deaths throughout the world with case fatality rates being highest in the developing countries [3]. Despite the increased educational and monetary investments by various public and private sector interest groups to improve outcomes, breast cancer still remains the second most significant cause of cancer-related mortality in the US population [4]. Per the 2002 guidelines by the National Cancer Control Program set forth by the World Health Organization, early screening, detection along with adequate therapy has been singled out as the most important factors in the fight for reduction in breast cancer mortality [5]. This is the basis of our 5-year retrospect cohort study. We looked at a variety of clinico-pathologic and demographic characteristics between the triple negative breast cancer (TNBC) and non-TNBC subtypes at our inner city program to compare our findings with some of the research studies published elsewhere.

## Methods

We reviewed data in our tumor registry between January 2000 and December 2005 with particular attention to mean age, race, family history, tobacco use and stage at presentation. We found a total of 445 patients with various subtypes of breast cancers. We included only those patients in whom the status of both estrogen and progesterone receptors (ER/PR) and the status of human epidermal growth factor 2 receptor (Her2/neu) protein overexpression were recorded. Our strict selection criteria lead to an exclusion of about 103 patients. Out of the remaining 342 patients, 39 were TNBC and 303 were non-TNBC.

## Results

Mean age of onset for TNBC vs. non-TNBC patients was 59.87 ± 15.67 years vs. 60.09 ± 13.98 years respectively (P = 0.9272). In terms of ethnicity, TNBC vs. non-TNBC patients had the following racial backgrounds: black, 58.97% vs. 39.27%; white, 35.90% vs. 57.76%; Chinese, 2.56% vs. 0.99%; others, 2.57% vs. 1.98% respectively (P = 0.004, OR = 2.755). Comparisons with respect to a history of tobacco abuse for TNBC vs. non-TNBC patients revealed a positive smoking history in 20.51% vs. 27.72% whereas there was no former or current smoking history in 71.79% vs. 61.72% respectively (P = 0.4385). Comparison of family history of a breast cancer in TNBC vs. non-TNBC patients showed that positive family history of breast cancer was seen in 30.77% vs. 33.33%, no family history of cancer was seen in 51.28% vs. 51.82% and unknown 17.95% vs. 14.85% (P = 0.8384). Pathologic stage at the time of diagnosis for TNBC vs. non-TNBC patients was as follows: stage 0, 15.79% vs. 11.37% (P = 0.4332); stage 1, 34.21% vs. 30.98% (P = 0.6890); stage 2, 28.98% vs. 37.25% (P = 0.3205); stage 3, 18.42% vs. 17.25% (P = 0.0.8591); and stage 4, 3.63% vs. 3.14% (P = 0.8651). Analysis using Chi-square test revealed χ^2^ value of 0.855. These findings are summarized in Table 1.

**Table 1:** Comparison of Demographics and Clinico-pathologic Characteristics in TNBC and Non-TNBC Patients

Possible risk factors	TNBC (%)	Non-TNBC (%)	
Mean age (years)	59.87 ± 15.67	60.09 ± 13.98	P = 0.9272
Ethnicity			P = 0.004; OR = 2.755
Black	58.97	39.27	
White	35.90	57.76	
Chinese	2.56	0.99	
Others	2.57	1.98	
Smoking history (past or present)			
Yes	20.51	27.72	P = 0.4385
No	71.79	61.72	
Family history			
Yes	30.77	33.33	P = 0.8384
No	51.28	51.82	
Unknown	17.69	14.85	
Stage at diagnosis			
Stage 0	15.79	11.37	P = 0.4332
Stage 1	34.21	30.98	P = 0.6890
Stage 2	28.98	37.25	P = 0.3205
Stage 3	18.42	17.25	P = 0.8591
Stage 4	3.63	3.14	P = 0.8651
Chi-square test (χ^2^)		0.855	

## Discussion

World Health Organization classifies breast cancer on the basis of histopathologic characteristics [6]. While this method successfully delineates breast cancer into several invasive subtypes, it fails to predict prognosis and treatment possibilities [7]. Recent advancements in the techniques used for immuno-histochemical and gene expression studies have lead to a distinct subdivision of breast cancer on the basis of protein expression and molecular subtypes respectively [8-11]. These newer techniques have resulted in the appreciation of breast cancer on the basis of expression for HER2/neu proteins and ER and PR [12]. Breast cancer classification, on the basis of receptors and proteins, has lead to the recognition of TNBC. It is classified as a type of breast cancer that lacks expression for ER and PR and does not over express Her2/neu proteins [13-15].

Due to its unique pathologic and clinical features, ranging from younger age at onset, higher propensity for distant visceral metastasis, poor outcomes and a more aggressive overall presentation, TNBC or tumor negative breast cancer has recently become the focal of intense breast cancer research [16]. TNBC subtype generally carries a worse prognosis when compared to its non-TNBC counterparts; however, it responds very well to the neo-adjuvant and adjuvant chemotherapies and conversely carries a much favorable response with these therapies [17]. TNBC has a higher predilection for certain ethnicities which is why its incidence has ranged from 11.2% in studies with a predominantly white patient population to as high as 39% in studies with a larger proportion of African American patients [18, 19]. In the Western world, the general consensus among the oncology community at large puts the prevalence of TNBC at around 15-20% [20, 21]. Based on our research, the prevalence of TNBC at our institution is 12.87%. While this percentage is lower than the generally agreed upon frequency of “15-20%”, we can attribute our lower prevalence to the diverse demographics in our sample population.

Irrespective of the breast cancer subtypes, the median age of patients at the time of affliction is also considered an important prognostic factor [22]. There are certain differences between the age of onset and the various breast cancer subtypes. TNBCs tend to occur at an earlier age compared to the more advances age of onset seen in the non-TNBC [23]. Since TNBC subtype has only recently been recognized as a distinct entity, it is not well understood if the prognosis differs between patients who developed TNBC at a younger age compared to those women who developed it at an older age [23]. Similarly the research data available are not conclusive enough to make a convincing argument for or against a biologic or clinical difference in TNBC patients by age at diagnosis [23]. The sparse research data available on breast cancer in general have shown variable results with some making a strong case for age as a distinct prognostic factor for breast cancer in younger patients whereas others failing to support this relationship [24, 25]. Our research study adds further statistical analysis to this growing body of evidence. We found that at our inner city university program, there was no difference in mean age at the time of diagnosis (P = 0.9272) between TNBC and non-TNBC patients.

Breast cancer subtypes also have a strong predilection with certain ethnic backgrounds. Data pooled from several research studies have indicated that African American women are more likely to have TNBC subtype than white women [26-31]. At our inner city institute, we found a significant statistical difference between the various ethnicities and their relationship with the two breast cancer subtypes (P = 0.004). Our retrospect research study indicated that black women were more likely to have TNBC, whereas the non-TNBC was more prevalent in white women.

Several *in vivo* and *in vitro* studies have shown that cigarette smoking has carcinogenic properties and the breast cancer tissue is a potential target for these carcinogens [32]. While the mechanism of action is not entirely understood, it is believed that the carcinogens in cigarette smoke are transported by the plasma lipoproteins on their journey from the alveoli to the breast tissue [33, 34]. Because of cigarette smoke’s strong affinity for these lipoproteins, it is more likely to be stored in adipocytes in the breast tissue which can later be activated by the human mammary epithelial cells to unleash its carcinogenic effect [35]. In cigarette smokers, the number of cigarette smoke based DNA adducts are significantly higher than in non-smokers [36-38]. Furthermore, researchers also point to the higher accumulation of P53 gene mutations in breast cancer tumors of smokers compared with non-smokers, which is also comparable to the mutational spectrum seen in lung cancer patients [32]. Besides the aforementioned biologic explanations, cigarette smoke is also thought to have an anti-estrogenic effect, supported by the observation that cigarette smokers have a lower bone density, an earlier age at menopause, decreased urinary levels of estrogens and an attenuated response to the hormone replacement therapy compared to non-smokers [39-42]. Ironically, the same cigarette smoke which is considered a risk factor for breast cancer can also have a protective role against breast cancer due to its anti-estrogenic effect [43]. With both a detrimental as well as a beneficiary profile, it is not difficult to imagine why several research studies, published previously, have shown inconsistent results about the relationship between cigarette smoking and breast cancer [44]. More recent research studies, however, have suggested a strong correlation between breast cancer in long-term cigarettes smokers and those who smoked before the birth of their first child [45-49]. In our research, there was no significant association between smoking status and cancer subtypes (P = 0.4385) which is in agreement with some of the earlier studies mentioned above.

In our research study, we also looked into family history and its relationship with TNBC and non-TNBC patients. About 10% of women with breast cancer have a positive family history of breast cancer [50]. A positive family history of breast cancer in a first-degree relative increases the risk of breast cancer by as much as two folds [50-52]. Both breast and ovarian cancers in the first-degree relatives are considered an established risk factor for the development of breast cancer [51]. Besides the prognostic significance, a positive family history is also associated with improved compliance with the early detection strategies like the mammography screenings [53-56]. Women with a positive family history are more likely to have fewer false beliefs about breast cancer and are likely to receive early breast cancer screenings and comprehensive breast cancer treatment [57, 58]. This might explain why, at our inner city program, we had a higher prevalence of breast cancer patients with a positive cancer history. Published studies have noted higher breast cancer mortality in women who have lower participation rates in mammography screening programs [59]. This further underscores the significance and future implications for patients who have a family history significant for another relative with breast cancer. At out inner city program, we found that there was no significant association between a positive family history of cancer in TNBC and non-TNBC subtypes (P = 0.8384). It is important to note that a positive family history of breast cancer does not impact all-cause mortality and a review of several published studies have failed to conclusively establish this relationship [53, 60-67]. Furthermore, BRCA-1 and BRCA-2 germ line mutations account for only a quarter of the total breast cancer cases and a significant portion of women with breast cancer acquire the affliction in the absence of this familial link [68].

Besides all the aforementioned risk factors, stage at diagnosis likely plays the most significant role in breast cancer mortality. Published research data by the National Cancer Institute have shown that the 5-year survival rate among women diagnosed with breast cancer at stage 1 is as high as 88% compared with the dismally low survival rate of around 15% for patients diagnosed with breast cancer at stage 4 [69, 70]. Non-Hispanic whites and Asians are more likely to be diagnosed at an earlier stage compared to Hispanic and black women who are likely to be diagnosed at an advanced stage [69, 70]. In our retrospect cohort research study, when accounting for stage at the time of diagnosis, TNBC was as prevalent as non-TNBC at all stages with P values of 0.4332, 0.6890, 0.3205, 0.8591 and 0.8651 for stage 0, 1, 2, 3 and 4 respectively. We found no significant difference in the stage at diagnosis in TNBC compared with the non-TNBC patients in our patient population.

### Conclusions

Our findings further contribute to the growing body of evidence pertaining to the association of certain demographic and clinico-pathologic characteristics in women with TNBC and non- TNBC. We found that in our patient population, there is a significant ethnic predisposition for these two subtypes of breast cancers. African Americans were more likely to have TNBC compared to the higher frequency of non-TNBC in white females. We did not find a significant difference in mean age, cigarette smoking, family history and stage at diagnosis between the TNBC and non-TNBC patients. These findings are all consistent with the previously published research studies.
